# DB-1310, an ADC comprised of a novel anti-HER3 antibody conjugated to a DNA topoisomerase I inhibitor, is highly effective for the treatment of HER3-positive solid tumors

**DOI:** 10.1186/s12967-024-05133-7

**Published:** 2024-04-17

**Authors:** Xi Li, Jun Yao, Chen Qu, Lan Luo, Bing Li, Yu Zhang, Zhongyuan Zhu, Yang Qiu, Haiqing Hua

**Affiliations:** Department of Research and Development, Duality Biologics, LTD, Unite 1106 868 Yinghua Road, Shanghai, 201204 P.R. China

**Keywords:** HER3, Antibody-drug conjugate, Solid tumor therapy, Preclinical

## Abstract

**Background:**

HER3 (ErbB3), a member of the human epidermal growth factor receptor family, is frequently overexpressed in various cancers. Multiple HER3-targeting antibodies and antibody–drug conjugates (ADCs) were developed for the solid tumor treatment, however none of HER3-targeting agent has been approved for tumor therapy yet. We developed DB-1310, a HER3 ADC composed of a novel humanized anti-HER3 monoclonal antibody covalently linked to a proprietary DNA topoisomerase I inhibitor payload (P1021), and evaluate the efficacy and safety of DB-1310 in preclinical models.

**Methods:**

The binding of DB-1310 to Her3 and other HER families were measured by ELISA and SPR. The competition of binding epitope for DB-1310 and patritumab was tested by FACS. The sensitivity of breast, lung, prostate and colon cancer cell lines to DB-1310 was evaluated by in vitro cell killing assay. In vivo growth inhibition study evaluated the sensitivity of DB-1310 to Her3 + breast, lung, colon and prostate cancer xenograft models. The safety profile was also measured in cynomolgus monkey.

**Results:**

DB-1310 binds HER3 via a novel epitope with high affinity and internalization capacity. In vitro, DB-1310 exhibited cytotoxicity in numerous HER3 + breast, lung, prostate and colon cancer cell lines. In vivo studies in HER3 + HCC1569 breast cancer, NCI-H441 lung cancer and Colo205 colon cancer xenograft models showed DB-1310 to have dose-dependent tumoricidal activity. Tumor suppression was also observed in HER3 + non-small cell lung cancer (NSCLC) and prostate cancer patient-derived xenograft (PDX) models. Moreover, DB-1310 showed stronger tumor growth-inhibitory activity than patritumab deruxtecan (HER3-DXd), which is another HER3 ADC in clinical development at the same dose. The tumor-suppressive activity of DB-1310 synergized with that of EGFR tyrosine kinase inhibitor, osimertinib, and exerted efficacy also in osimertinib-resistant PDX model. The preclinical assessment of safety in cynomolgus monkeys further revealed DB-1310 to have a good safety profile with a highest non severely toxic dose (HNSTD) of 45 mg/kg.

**Conclusions:**

These finding demonstrated that DB-1310 exerted potent antitumor activities against HER3 + tumors in in vitro and in vivo models, and showed acceptable safety profiles in nonclinical species. Therefore, DB-1310 may be effective for the clinical treatment of HER3 + solid tumors.

**Supplementary Information:**

The online version contains supplementary material available at 10.1186/s12967-024-05133-7.

## Background

HER3 is a member of the EGFR family [1]. Its overexpression has been found in many solid tumors, including breast, head and neck, lung, colorectal, prostate and ovarian cancers [2]. High HER3 expression is also linked to disease progression and poor prognosis in many cancer types [3]. Due to its common expression in numerous tumors and efficient cancer cell internalization upon antibody binding, HER3 has become a feasible target especially for ADC development [4].

Although HER3 is a compelling target for cancer therapy, no HER3-targeting therapy has been approved for clinical use. Patritumab deruxtecan (HER3-DXd; U3-1402) is the HER3-targeting ADC in clinical development and shown clinical benefit in breast cancer and NSCLC patients with or without EGFR mutations [5, 6]. It had an objective response rate (ORR) of 45% in heavily pretreated metastatic breast cancer patients in a phase 1/2 study [7, 8]. EGFR tyrosine kinase inhibitors (EGFR-TKIs) were approved for first-line therapy in advanced NSCLC patients harboring EGFR mutation [9]. Resistance to EGFR-TKIs eventually develops after therapy. In a phase II study in metastatic EGFR-mutant (EGFRm) and EGFR-TKI-resistant NSCLC patients, HER3-DXd led to a response rate of 39% [10]. More importantly, clinical efficacy was observed in NSCLCs exhibiting diverse EGFR TKI resistance mechanisms, as HER3 overexpression is not a known resistance mechanism to EGFR-TKIs [5, 11–13]. Those most recent clinical advancement suggests that HER3 is a promising oncology target. However, hematologic toxicities, including thrombocytopenia and neutropenia, were the most common treatment-emergent adverse events observed during the clinical development of HER3-DXd [5, 10]. Therefore, development of novel HER3-targeting ADC drugs remains a great unmet need.

ADC are a class of anticancer agents consist of a cytotoxic drug (called a ‘payload’) conjugated via a linker to a monoclonal antibody (mAb) targeting an antigen of interest that is specifically expressed on cancer cells. The cytotoxic payload varies, ranging from microtubule inhibitors to deoxyribonucleic acid cleavage agents and topoisomerase inhibitors [14]. The mechanism of ADCs is typically thought to begin with the binding of the mAb to the target antigen, leading to internalization, linker breakdown and release of the cytotoxic payload. Multiple factors affect the cytotoxicity and safety profile of the ADC molecule such as the binding epitope of antibody, payload type, the drug-antibody ratio (DAR), linker-payload stability and the hydrophobicity of the detached payload. The selection of antibody with efficient up-taken by antigen-bearing cells and payload with efficient cytotoxicity and manageable safety profile would be essential for the development of ADC drug.

ADCs containing topoisomerase inhibitors, such as trastuzumab deruxtecan (DS-8201), which was developed for cancer therapy, have been successfully used in various cancer types [15, 16]. Despite its efficiency in the clinic, therapies with topoisomerase-directed agents are limited by its dose-limiting toxicities. Interstitial lung disease is an adverse event observed during clinical therapy with DS-8201 [17]. Accordingly, development of effective topoisomerase inhibitor payload to treat cancers with fewer adverse effects are still urgently desirable.

In this study, we developed DB-1310, an anti-HER3 ADC conjugating with a novel HER3 antibody and proprietary DNA topoisomerase I inhibitor payload (P1021), investigated its mechanism of action and preclinically evaluated its efficacy in breast, NSCLC, colon and prostate cancer models. DB-1310 demonstrated HER3-dependent up-taken by cells and antitumor activity both in vitro and in vivo. It also showed synergistic antitumor effect with an EGFR inhibitor, osimertinib, in the NSCLC and suppressed growth of PDX from osimertinib resistant NSCLC patient.

## Methods

### Cell lines

The A549, Calu-6, Colo205, DU-145, HCC1569, HEK293, JIMT-1, MDA-MB-231, MDA-MB-453, NCI-H441, NCI-H1569, NS-1, PC-3, SK-BR-3, SP2/0 and 22RV-1 cell lines used were purchased from ATCC and maintained in the recommended medium. HEK293-ERBB3 cells were purchased from Kyinno Biotechnology (KC-1496). SP2/0-HER3 cells stably expressing human HER3 were generated in house by transduction of human HER3 with lentivirus construct.

### Development of the anti-HER3 mAb

BALB/c mice (female, aged 8–10 weeks) were intraperitoneally injected with 1-2 × 10^6^ SP2/0-HER3 cells expressing human HER3 together with Freund’s complete adjuvant. On days 8, 14, 17 and 20, mice were immunized with the abovementioned number of cells and Freund’s incomplete adjuvant. On day 23, splenic B lymphocytes were isolated from the mice and fused with immortalized myeloma NS-1 cells to generate hybridoma cells. Animals were maintained and used according the guidelines of IACUC. The generated hybridoma cells were cultured in a 96-well plate containing HAT Media Supplement (Sigma-Aldrich, Saint Louis, MO). The supernatants were collected to screen for antibodies that recognize surface HER3 on SP2/0 cells by flow cytometry and for antibodies that recognize recombinant HER3 by ELISA. Antibody clone 3f8, which specifically binds human HER3, was selected, and the DNA sequences encoding the antibody heavy chain and light chain were inserted into the expression vector pCDNA3.1(+) (Invitrogen, Carlsbad, CA). The antibody was then humanized as previously described [18]. In brief, the mouse residues that are essential for maintaining affinity and specificity were preserved, whereas the other mouse residues were replaced with the corresponding human germline residues (Supplementary Fig. 1). The DNA sequences encoding the humanized 3f8 antibody (Hu3f8) were synthesized by GenScript and expressed in 293T cells.

### Flow cytometric analysis

Tumor cells were digested with trypsin and counted to ensure that the viability was > 90.0% by trypan blue staining. The harvested cells were washed twice with FACS staining buffer (Thermo Fisher Scientific, Carlsbad, CA 00-4222) and stained with 100 nM anti-HER3 antibody or human IgG1 isotype for 60 min at 4 °C. The cells were washed twice with FACS staining buffer and stained with a PE-conjugated anti-human IgG Fc antibody (1:500, BioLegend, San Diego, CA. 410,708) for 30 min at 4 °C. Fluorescence data were collected using a Fortessa X20 flow cytometer (Becton and Dickinson Company) and analyzed using FlowJo (BD Biosciences, San Jose, CA, USA).

### ELISA

Recombinant human HER2, HER3, HER4, and EGFR and mouse HER3 were purchased from ACRO Biosystems, Inc. Recombinant cynomolgus HER3 and rat HER3 were purchased from Sino Biological, Inc. ELISA plates were coated with 1 µg/mL recombinant protein in PBS at 4 °C overnight and incubated with DB-1310, Hu3f8 or human IgG1 in serial dilutions starting at 100 nM. Anti-human IgG-peroxidase (1:5000, Sigma-Aldrich, St Louis, MO) was used as the secondary antibody for detection of the TMB signal (Biopanda Diagnostics, Belfast, UK TMB-S-003) at 450 nm in a microplate reader (Molecular Devices, San Jose, CA). GraphPad Prism 6.0 software was used for data analysis.

### Internalization and trafficking assays

SK-BR-3 and NCI-H441 cells were seeded into 96-well plates or confocal dishes and cultured overnight. DB-1310 and HER3-DXd were labeled with Zenon pHrodo iFL IgG labeling reagents (Thermo Fisher Scientific, Inc.). The cells were incubated with the pHrodo-labeled complex (1 nM) for up to 48 h under standard cell culture conditions at 37 °C. At each detection time point, nuclei were stained with Hoechst 33342, and the fluorescence signal was detected by an Operetta CLS high-content analytical system. The intracellular localization of the pHrodo-labeled complex was tracked by incubating cells with LysoTracker and Hoechst 33342 and visualizing fluorescence by Leica TCS 8MP multiphoton confocal microscope (Leica microsystems).

### Epitope binning

MDA-MB-453 cells were seeded into 96-well plates overnight. Serial dilutions of patritumab or Hu3f8 starting at 500 nM were prepared with staining buffer (PBS + 1% FBS) and mixed with 10 nM biotin-labeled patritumab. The cells were stained with the antibody mixtures for 1 h at 4 °C, and biotin labeling was detected with streptavidin-PE (1:5000, BD Biosciences, San Diego, CA). The fluorescence signal was measured by flow cytometry. The inhibition rate was calculated as [1- (mean fluorescence intensity of 10 nM biotin-labeled patritumab - mean fluorescence intensity of the antibody mixture)].

### Cell cycle analysis

Cells were washed with cold PBS and resuspended in ice-cold PBS. The cells were fixed with 66% ethanol by adding ice-cold 100% ethanol slowly to the cell suspension. The cells were stored at 4 °C overnight, washed and collected by centrifugation (4 °C, 800 g, 10 min). The cell pellet was gently resuspended in 1x propidium iodide (PI)/RNase staining solution (BD Biosciences), and the samples were analyzed on a Fortessa X20 flow cytometer (BD Biosciences). The fluorescence signal of PI was measured by FACSDiva (BD Biosciences, San Jose, CA), and the intensities of the 2 N and 4 N peaks were plotted on a linear scale for calculation of the proportions of cells in each stage of the cell cycle.

### Bystander killing effect analysis

HEK293 cells were labeled with CellTrace Far Red Dye (Invitrogen) and mixed with an equal number of HEK293-ERBB3 cells. The mixed cells were seeded in 96-well flat-bottom plates, and DB-1310, Hu3f8, HER3-DXd or human IgG1 isotype control was added to a final concentration of 0.1 nM or 0.5 nM. The viability of both HEK293 and HEK293-ERBB3 cells was determined by PI staining and flow cytometric analysis. The data were processed with FlowJo (BD Biosciences, San Jose, CA, USA), and the proportion of the PI + subpopulation was determined.

### Antibody-dependent cell-mediated cytotoxicity (ADCC) assay

PBMCs and CellTrace™ Far Red Dye-labeled HEK293-ERBB3 target cells were cocultured at various effector: target (E: T) ratios to determine the optimal ratio to use in the assay. DB-1310 or Hu3f8 was added to the cells at a final concentration of 15 µg/ml. The plate was incubated at 37 °C for 6 h, and the lysis rate of HEK293-ERBB3 cells was determined by PI (Invitrogen) staining and flow cytometric analysis. The appropriate E: T ratio of 20:1 was determined based on the 80% maximum cell lysis at this ratio. PBMCs and HEK293-ERBB3 target cells were cocultured at this ratio for ADCC detection. DB-1310 or Hu3f8 was added to the cell coculture at a series of concentrations starting from 15 µg/ml. The effect of DB-1310 or Hu3f8 on target cell lysis was quantified based on the HEK293-ERBB3 cell lysis rate.

### Complement-dependent cytotoxicity (CDC) assay

HEK293-ERBB3 cells were suspended in culture medium and seeded into a 96-well U-bottom plate. DB-1310 or Hu3f8 was added to the wells at a series of concentrations. Normal human serum complement (Quidel San Diego, CA) was added to the corresponding wells to a final concentration of 2.5%. The cells were cultured at 37 °C for 1 h. The CytoToxi96® Non-Radioactive Cytotoxicity Assay Kit (Promega) was used to detect CDC-mediated lysis following the manufacturer’s protocol.

### In vitro tumor suppression assay

Cells were seeded at 1000 to 4000 cells per well in 96-well plates overnight. DB-1310 was diluted with complete medium and added to the cells at different concentrations starting from 1000 nM and progressing with 3-fold dilutions. The cells were cultured for 7 days without medium replacment, and cell viability was measured using a CellTiter-Glo Luminescent Cell Viability Assay (Promega) following the manufacturer’s instructions. The inhibition rates observed at different DB-1310 concentrations were calculated, and the half-maximal inhibitory concentration (IC50) was interpreted from the dose‒response curve using Graphpad Prism (GraphPad, Boston, MA).

### Western blotting

NCI-H441 tumor samples from xenograft were homogenized with RIPA lysis buffer (Thermo Fisher Scientific) supplemented with protease inhibitor and phosphatase inhibitor. The lysate was centrifuged, and the supernatant was collected for quantification of the protein concentration by a BCA assay (Thermo Fisher Scientific). Equal amounts of proteins from tumor lysates were separated via SDS‒PAGE, and the separated proteins were transferred to nitrocellulose membranes. Immunoblot analysis of p-γ-H2AX (Cell signaling technology, Beverly, MA) and β-actin (Cell signaling technology, Beverly, MA) was performed. The primary antibodies and HRP-conjugated secondary antibody were purchased from Cell Signaling Technology, Inc. The membranes were incubated with HRP substrate, and immunoreactions were detected using a Tanon 5200 chemiluminescence image analysis system. Band densities were quantified using ImageJ.

### Xenograft studies

Cell line-derived xenograft (CDX) and patient-derived xenograft (PDX) models were established in immunocompromised NU/NU mice for NCI-H441 and LD1-0025-200717 model, NOD/SCID mice for HCC1569 model, BALB/c nude mice for LU1542, NCI-H1975 and Colo205 mice, NPG mice for PR9586 model or NOG mice for PR9587 model. When the tumor volume reached approximately 100–200 mm^3^, the tumor-bearing mice were randomly assigned to control and treatment groups for 6–8 animal per group. DB-1310 was administered via either single intravenous injection or biweekly at a dose of 1 to 10 mg/kg for 4 weeks. Osimertinib (1 mg/kg) was administered orally daily. Tumor diameters and body weights were measured twice a week. Tumor volume was calculated using the equation Tvol = 1/2 x length x width^2^. The tumor growth inhibition rate (TGI %) was calculated according to the formula 100x [1 – (average tumor volume in the treatment group)/(average tumor volume in the control group)]. Animals were euthanized by CO_2_ inhalation at the end of the experiments. All animal experiments performed in this study were approved by the Institutional Animal Care and Use Committee at Crownbio Co., Ltd.

### Toxicity studies in monkeys

DB-1310 was intravenously administered to cynomolgus monkeys at 15, 30 or 45 mg/kg once every 2 weeks for a total of 3 doses, followed by a 4-week recovery period. Clinical signs, body weight, food consumption, and clinical pathology were monitored throughout both treatment and recovery period. Necropsy was performed on the day after the recovery period ended.

## Results

### DB-1310 has high binding affinity for HER3 and internalizes to HER3 + cells

**Fig. 1:**
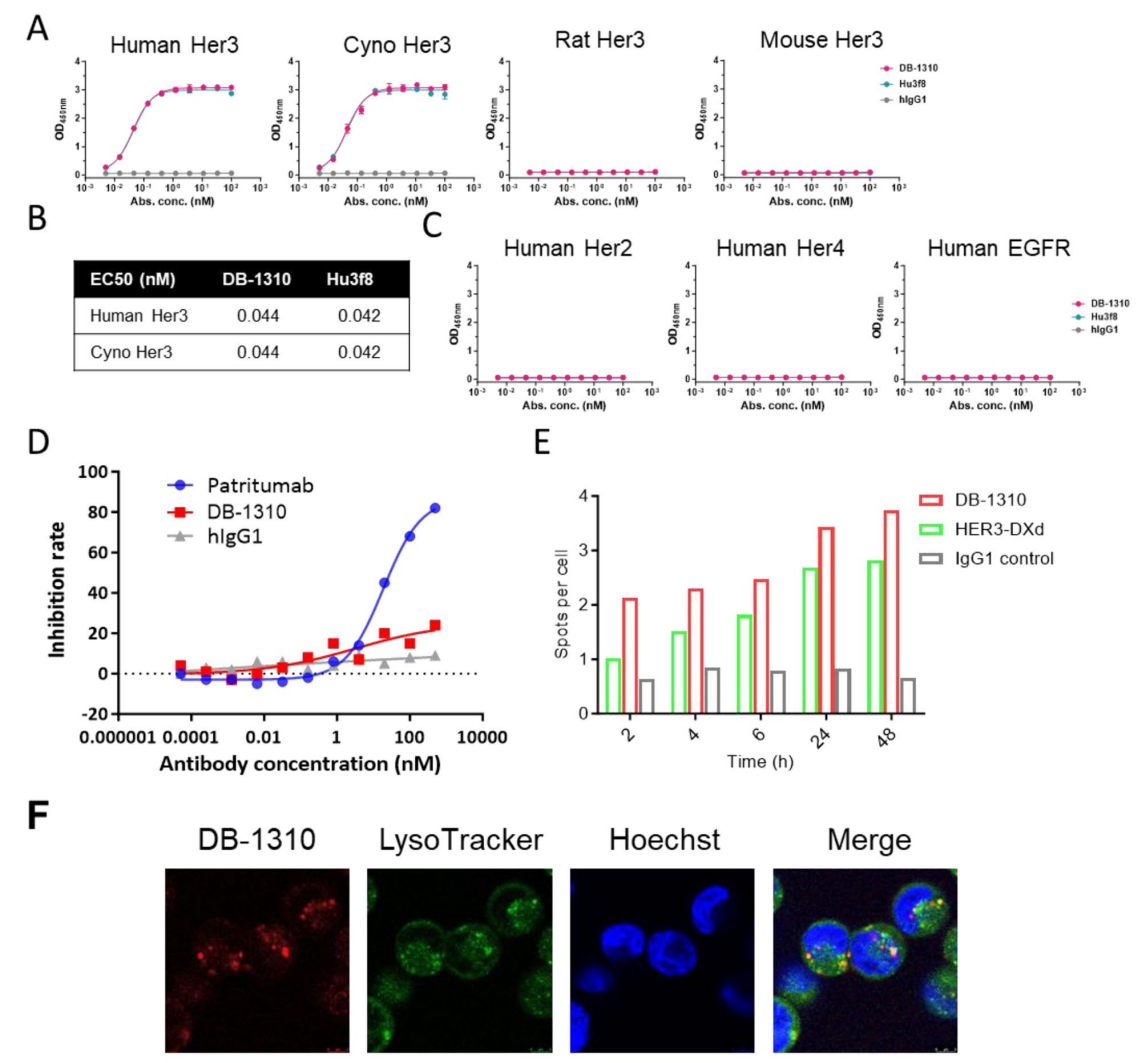
DB-1310 binds HER3 via a novel epitope with high affinity and internalization capability. (**a-c**) The binding of DB-1310 and its parental antibody to human HER3, cynomolgus HER3, human HER2, human HER4 and human EGFR was measured by ELISA. (**d**) MDA-MB-453 cells were stained with 10 nM biotin-labeled patritumab in the presence of DB-1310 or naked patritumab at serial dilutions of up to 500 nM. The binding of biotin-patritumab to cell surface HER3 was detected by streptavidin-PE staining. The Mean fluorescence intensity (MFI) of PE was measured for each condition, and the inhibition rate was calculated as (MFI _biotin−patritumab only_ – MFI _test condition_)/ MFI _biotin−patritumab only_ x 100%. (**e**) Internalization of pHrodo-labeled DB-1310 or HER3-DXd was measured by an Operetta CLS high-throughput microplate imager. (**f**) MDA-MB-453 cells were cultured with pHrodo-labeled DB-1310 for 12 h and images were obtained by confocal microscopy. Lysosomes and nuclei were labeled with LysoTracker (green) and Hoechst (blue), respectively

We developed a novel HER3 targeting ADC, DB-1310, and characterized its binding affinity, specificity, epitope and internalization capability in vitro. A humanized HER3 antibody, Hu3F8, was identified from the mouse hybridoma immunized with SP2/0 cells overexpressing human HER3. Hu3F8 recognizes human and monkey HER3 with a sub-nanomolar binding affinity as measured by ELISA (Fig. 1a and b). DB-1310 was generated by covalently linking Hu3f8 to a proprietary DNA topoisomerase I inhibitor payload (P1021) via a maleimide tetrapeptide-based cleavable linker [19]. This unique linker-payload system could increase hydrophilicity of ADC, achieving a high homogenous conjugation and high drug-to-antibody ratio. Hydrophobic interaction chromatography showed a homogenous drug distribution and a DAR of approximately eight (Supplementary Fig. 1). We tested binding affinity of DB-1310 to HER3 by ELISA and compared that with parental antibody. DB-1310 showed high binding affinity for both human and cynomolgus monkey HER3 with a half-maximal effective concentration (EC50) of 0.044 nM (Fig. 1a and b). The binding affinity of DB-1310 for human and cynomolgus monkey HER3 was the same as that of parental antibody (Fig. 1b). Neither DB-1310 nor Hu3f8 displayed binding activity toward murine HER3 (Fig. 1a). The high antigen binding affinity of DB-1310 was further confirmed by surface plasmon resonance (SPR) analysis. DB-1310 bound human and cynomolgus monkey HER3 with KD values of approximately 0.499 nM and 1.27 nM, respectively (Supplementary Table 1). These results showed that DB-1310 exhibits high HER3 binding affinity and the conjugation of the payload does not impair antibody binding.

Among the EGFR family receptors, Her2, Her3 and Her4 are closely related and share relatively high sequence and structure similarity [20]. We next assessed whether DB-1310 could cross react with other EGFR family members. In the ELISA, DB-1310 did not bind human Her2, Her4 or EGFR at concentrations of up to 100 nM (Fig. 1c). It showed a high selectivity against the other ERBB family members.

HER3-DXd exhibits high internalization efficacy and translocation to lysosomes [21]. The quickly and efficiently internalization by cells is essential for a desirable ADC drug. Therefore, we compared the epitope binding abilities and internalization dynamics of DB-1310 and patritumab in HER3 expressing MDA-MB-453 cells using human IgG as a control. DB-1310 bound HER3 without competing with biotin-labeled patritumab on the cell surface (Fig. 1d). The internalization and lysosomal localization of DB-1310 were visualized by a CLS high-content analytical system and immunofluorescence microscopy with pHrodo Red (an acidic pH sensitive probe) labeling. DB-1310 was efficiently internalized into cells and translocated into lysosomes with a higher internalization capability than that of HER3-DXd (Fig. 1e and f). Therefore, DB-1310 binds HER3 via a novel epitope from patritumab with high internalization capability.

### DB-1310 has antitumor activity in vitro and in vivo

**Fig. 2:**
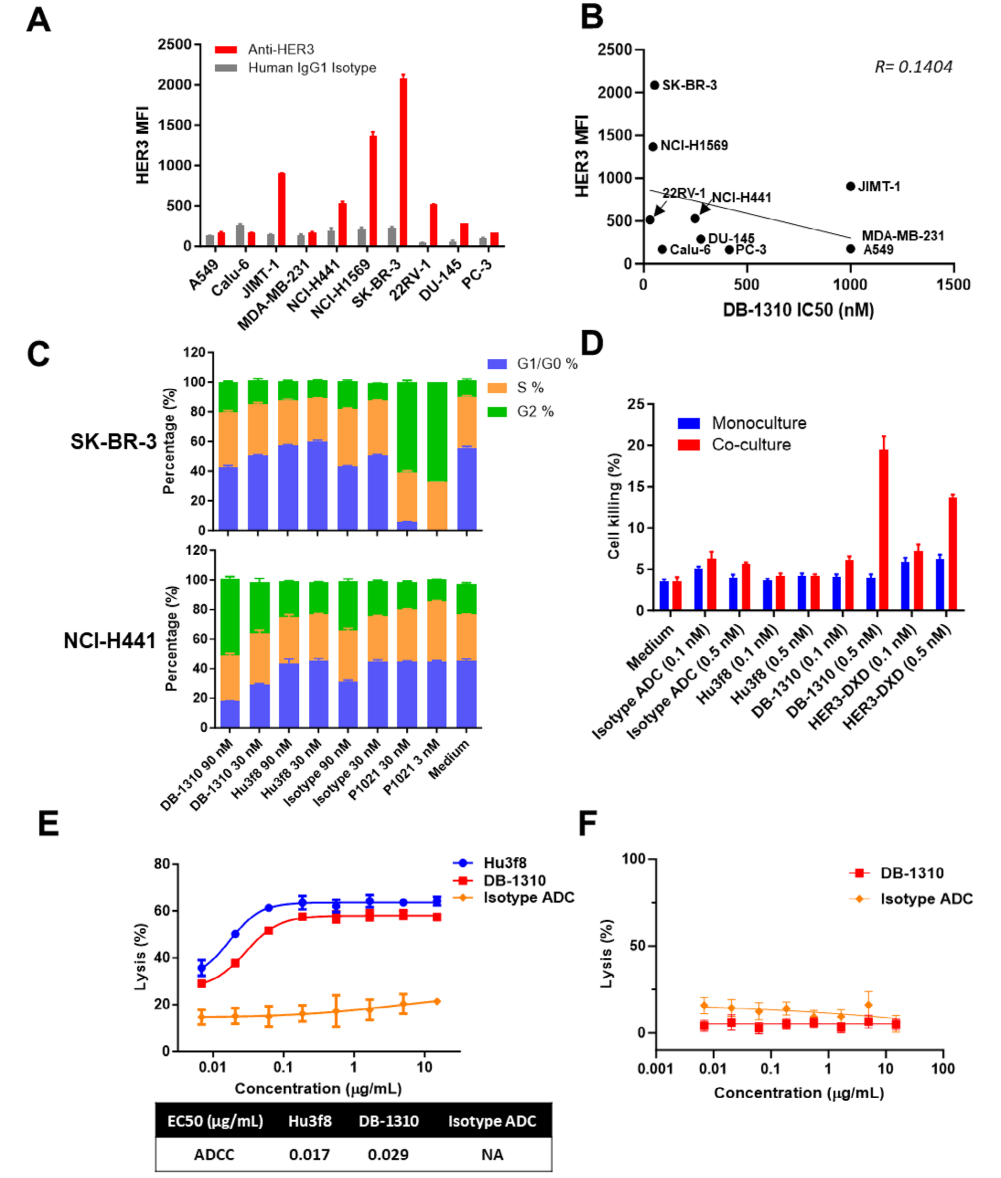
DB-1310 suppresses the growth of tumor cells in vitro. **(a)** Surface HER3 was detected by FACS in tumor cell lines. HER3 expression was determined as the MFI. **(b)** Breast cancer, prostate cancer and lung cancer cell lines were cultured with DB-1310. Cell proliferation was measured by a CTG assay, and IC50 values were calculated using linear regression with GraphPad Prism. **(c)** SK-BR-3 and NCI-H441 cells were treated with DB-1310 or Hu3f8 at 30 nM or 90 nM, and P1021 at 3nM or 30nM for 48 h. The cell cycle was analyzed by FACS. **(d)** HEK293 cells were stained with CellTrace™ Far Red Dye to trace in analysis and cocultured with HER3-negative HEK293 or HER3-expressing HEK293-ERBB3 cells at a 1:1 ratio for 4 days in the presence of different treatments. Cells were stained with PI, and labeled cell killing was analyzed by FACS. **(e)** HER3-expressing HEK293-ERBB3 cells were labeled with CellTrace™ Far Red Dye and cocultured with PBMCs at a 1:20 ratio in the presence of DB-1310 for 6 h to access ADCC effect. Cells were stained with PI and subjected to flow cytometric analysis to determine the lysis rate of HEK293-ERBB3 cells. (**f**) Human serum complement and HEK293-ERBB3 cells were mixed and incubated with DB-1310 for 1 h. Complement-mediated lysis of target cells was measured by CytoToxi96® Non-Radioactive Cytotoxicity Assay kit

Given that DB-1310 is quickly up-taken and translocated to lysosomal by HER3 + tumor cells, we next sought to determine its antiproliferative effect in tumor cell lines with different HER3 expression levels in vitro using a CellTiter-Glo Luminescent Cell Viability (CTG) assay. The P1021 payload markedly inhibited the proliferation of most tumor cells with IC50 values of 0.4-5 nM. The observed P1021-mediated inhibitory effect was not significantly correlated with the level of HER3 expression and the proliferative suppression was not observed in Hu3f8 treatment (Supplementary Fig. 2). DB-1310 inhibited proliferation in SK-BR-3, NCI-H441, NCI-H1569 and 22RV-1 cell lines which have high HER3 protein expression. DB-1310 had little inhibitory effect on the proliferation of JIM-1, MDA-MB-231, A549, DU-145 and PC-3 cells, which have low to no HER3 expression or are less sensitive to the small molecule toxin P1201. HER3-independent cell growth inhibition activity was observed in Calu-6 cells which have little cell surface HER3. This may contribute to its high sensitivity to P1201 and unspecific internalization (Fig. 2a and b and Supplementary Fig. 2). Collectively, our results imply that DB-1310 showed cytotoxicity activity to multiple tumor cells.

Inhibition of topoisomerase blocks DNA replication with G2/M cell cycle arresting of rapid-dividing tumor cells [22, 23]. The effect of DB-1310 and the payload on the cell cycle was assessed in HER3 + SK-BR-3 and NCI-H441 cells by flow cytometry because DB-130 significantly inhibited cell proliferation in these cell lines. P1021 and DB-1310, but not Hu3f8, induced G2/M arrest, indicating that this effect was mediated primarily by the payload P1021 (Fig. 2c).

P1021 is a cell membrane-permeable payload. It enables a bystander antitumor effect, resulting in the elimination of both target cells and surrounding tumor cells [19]. The bystander killing effect of DB-1310 was determined by comparison of cell killing in a monoculture of HER3-negative HEK293 cells and a coculture of HEK293 cells with HEK293-ERBB3 cells, a HER3-expressing 293T cell line. When used at 0.5 nM, DB-1310 inhibited the proliferation of HEK293 cells in the coculture system but not in the monoculture system, indicating that DB-1310 exerts a bystander killing effect (Fig. 2d).

The antitumor activity of antibody-based therapy could be also mediated through ADCC and CDC [24]. Both DB-1310 and Hu3f8 showed high binding affinity for FcγRI, FcγRIII and C1q (Supplementary Table 2), suggesting that DB-1310 may retain strong ADCC activity. We examined whether DB-1310 induces ADCC and CDC in HEK293-ERBB3 cells. Both DB-1310 and Hu3f8 exhibited substantial ADCC activity in HEK293-ERBB3 cells in the assay with PBMCs, with EC50 values of 0.029 and 0.017 µg/ml, respectively (Fig. 2e). In contrast, neither DB-1310 or IgG1 exhibited CDC activity in HEK293-ERBB3 cells (Fig. 2f). In summary, these in vitro findings indicated that DB-1310 may induce strong antitumor activity in vivo as well.

### DB-1310 has antitumor effects in mouse xenograft models

**Fig. 3:**
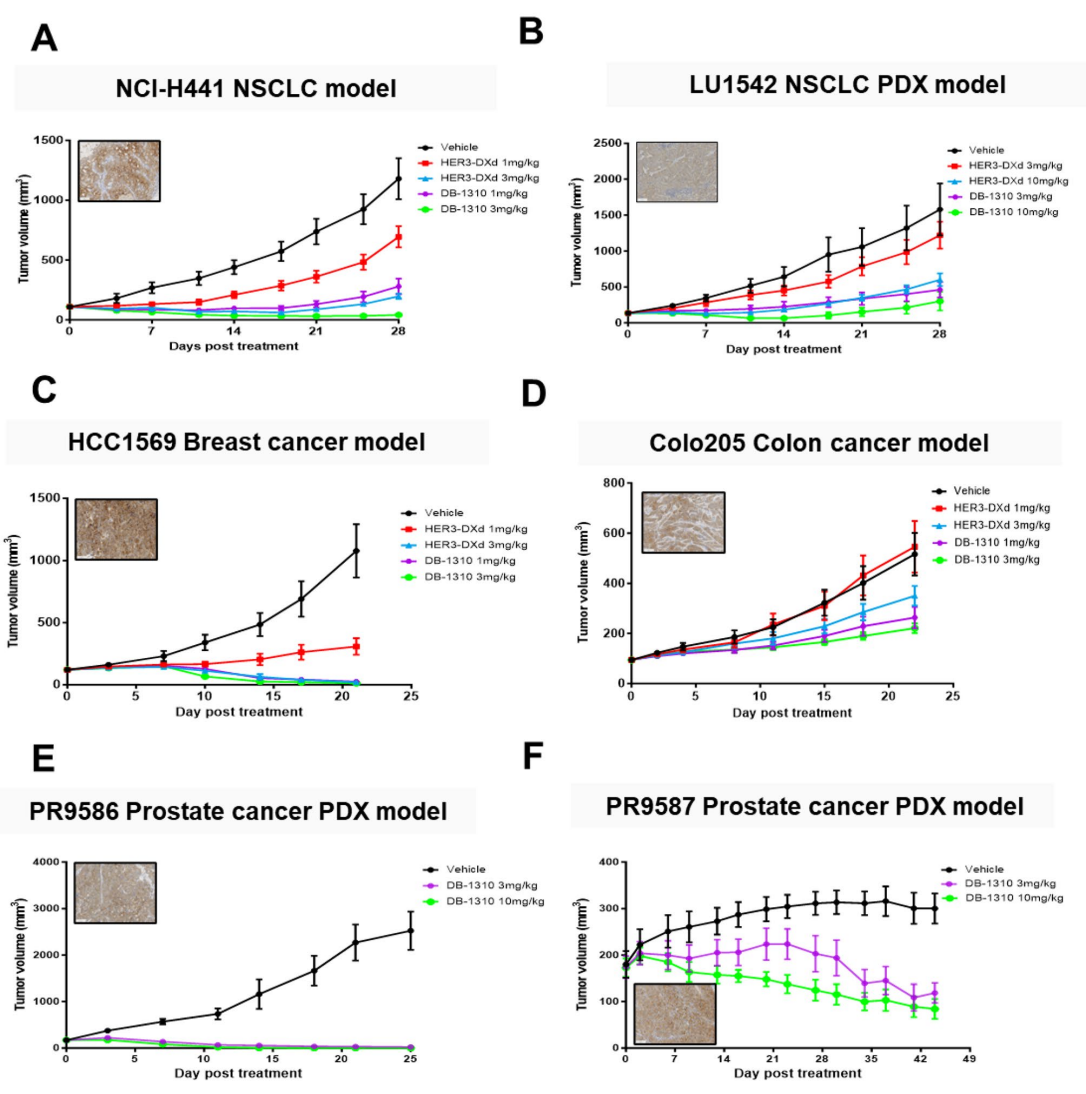
DB-1310 inhibits tumor growth in vivo. Immunodeficient mice were inoculated with NCI-H441 NSCLC cells (**a**), LU1542 NSCLC patient-derived tumor xenografts (**b**), HCC1569 breast cancer cells (**c**), Colo25 colon cancer cells (**d**), PR9586 prostate cancer patient-derived tumor xenografts (**e**) or PR9587 prostate cancer patient-derived tumor xenografts (**f**) and were then treated with DB-1310 or HER3-DXd. The tumor volume in each animal was measured twice a week

**Fig. 4:**
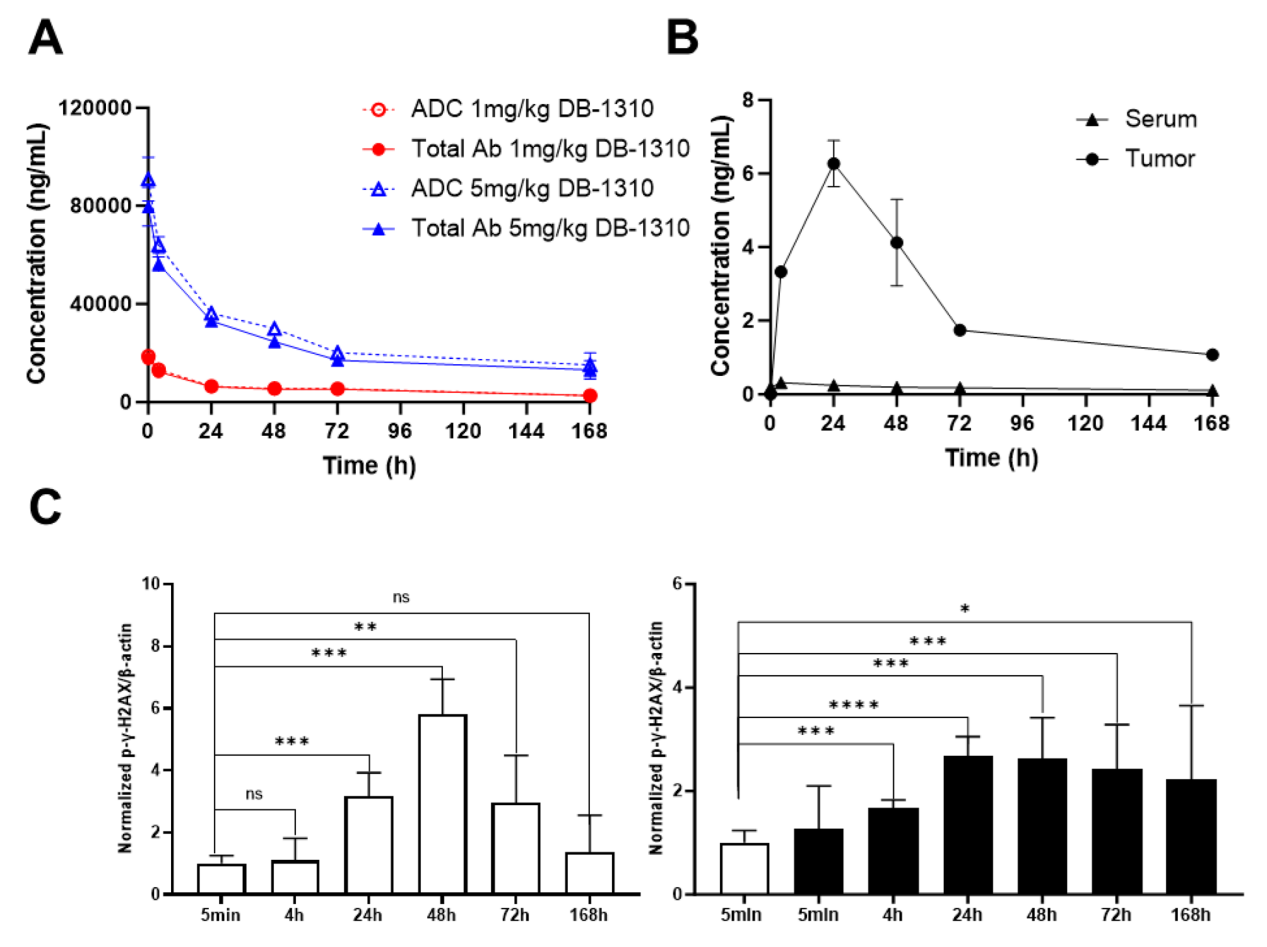
Tumor-specific release of P1021 payload contributes to DB-1310 in vivo efficacy in the NCI-H441 model. NU/NU mice were subcutaneously inoculated with 5 × 10^6^ NCI-H441 NSCLC cells and intravenously injected with either 1 or 5 mg/kg DB-1310. Serum and tumor samples were collected at different time points. Total antibody and ADC concentrations in serum were measured by ELISA (**a**). Free payload concentrations in serum and tumor samples were measured by LC-MS/MS (**b**). (**c**) The level of P-H2AX in tumors was measured by western blotting and normalized to β-actin

We evaluated in vivo antitumor activity of DB-1310 through CDX mouse models as well as PDX models. DB-1310 showed strong dose-dependent antitumor activity in the HER3-expressing NCI-H441 human lung cancer xenograft model and LU1542 lung cancer PDX models, demonstrating the dependence of HER3 expression on the tumor-suppressive activity of DB-1310 (Fig. 3a and b). Significant TGI induced by DB-1310 was demonstrated at 1 and 3 mg/kg in the NCI-H441 model and at 3 and 10 mg/kg in the LU1542 model comparing with vehicle control. The maximum TGI % was greater than 95% in the NCI-H441 model and greater than 80% in the LU1542 model. In addition, DB-1310 showed higher tumor-suppressive activity than HER3-DXd in both tested models, without affecting the body weight of any tumor-bearing animal, even at doses of up to 10 mg/kg. In addition to the lung cancer models, tumor growth was significantly suppressed in the DB-1310 groups compared with the vehicle groups in an HCC1569 breast cancer model, a Colo205 colon cancer model, and PR9586 and PR9587 prostate cancer PDX models (Fig. 4c-f). DB-1310 also showed dose-dependent antitumor activity with superior tumor-suppressive activity compared with that of HER3-DXd in both HCC1569 and Colo205 models. Collectively, these results indicated that DB-1310 reduced the growth of several diverse HER3-expressing tumors.

### The in vivo efficacy of DB-1310 is due to the tumor-specific release of P1021 payload

We further explored the therapeutic mechanism of DB-1310 through in vivo pharmacokinetic/ pharmacodynamic (PK/PD) study in the NCI-H441 human lung cancer xenograft model by treatment with a single intravenous injection of 1 or 5 mg/kg DB-1310. The DB-1310-ADC, total antibody and free payload concentrations in serum and tumor samples were measured by ELISA or LC-MS/MS respectively. The serum and tumor free payload concentrations were below the detection limit after administration of 1 mg/kg DB-1310. The area under the curve (AUC) and Cmax of serum DB-1310-ADC or DB-1310-total antibody increased in a dose-dependent manner (Fig. 4a). Moreover, in the 5 mg/kg DB-1310 group, the ratio of the AUC of P1021 to that of DB-1310-ADC was approximately 111-fold higher in the tumor than in serum (Fig. 4b; Table 1), implying that P1021 was effectively released in the tumor. The level of the DNA damage marker phosphorylated Histone 2AX (P-H2AX) in tumors increased in a time-dependent manner from 24 to 72 h after drug administration in the DB-1310 1 mg/kg group, peaking at 48 h. In the 5 mg/kg DB-1310 group, the P-H2AX level was increased at 4 h and remained high until 168 h (Fig. 4c and Supplementary Fig. 2). These results implied that P1021 was effectively released in tumors, which led to high exposure to P1021 in tumors but low exposure to P1021 in serum and potentially accounts for the high efficacy and low toxicity of DB-1310.

**Table 1:** PK parameters of DB-1310 (5 mg/kg, i.v.) in the NCI-H441 model

Tissue	Analyte	T_max_	t_1/2_	C_max_	AUC_0 − t_
		h	h	ng/mL	ng.h/mL
**Serum**	ADC	0.0830	119	90,996	4,355,036
	Total antibody	0.0830	118	79,688	3,766,065
	P1021	4.00	144	0.318	29.8
**Tumor**	ADC	48.0	88.4	5021	558,242
	Total antibody	48.0	87.8	4544	500,316
	P1021	24.0	59.9	6.27	424

### DB-1310 and osimertinib showed synergistic antitumor activity against EGFRm NSCLC xenografts

**Fig. 5:**
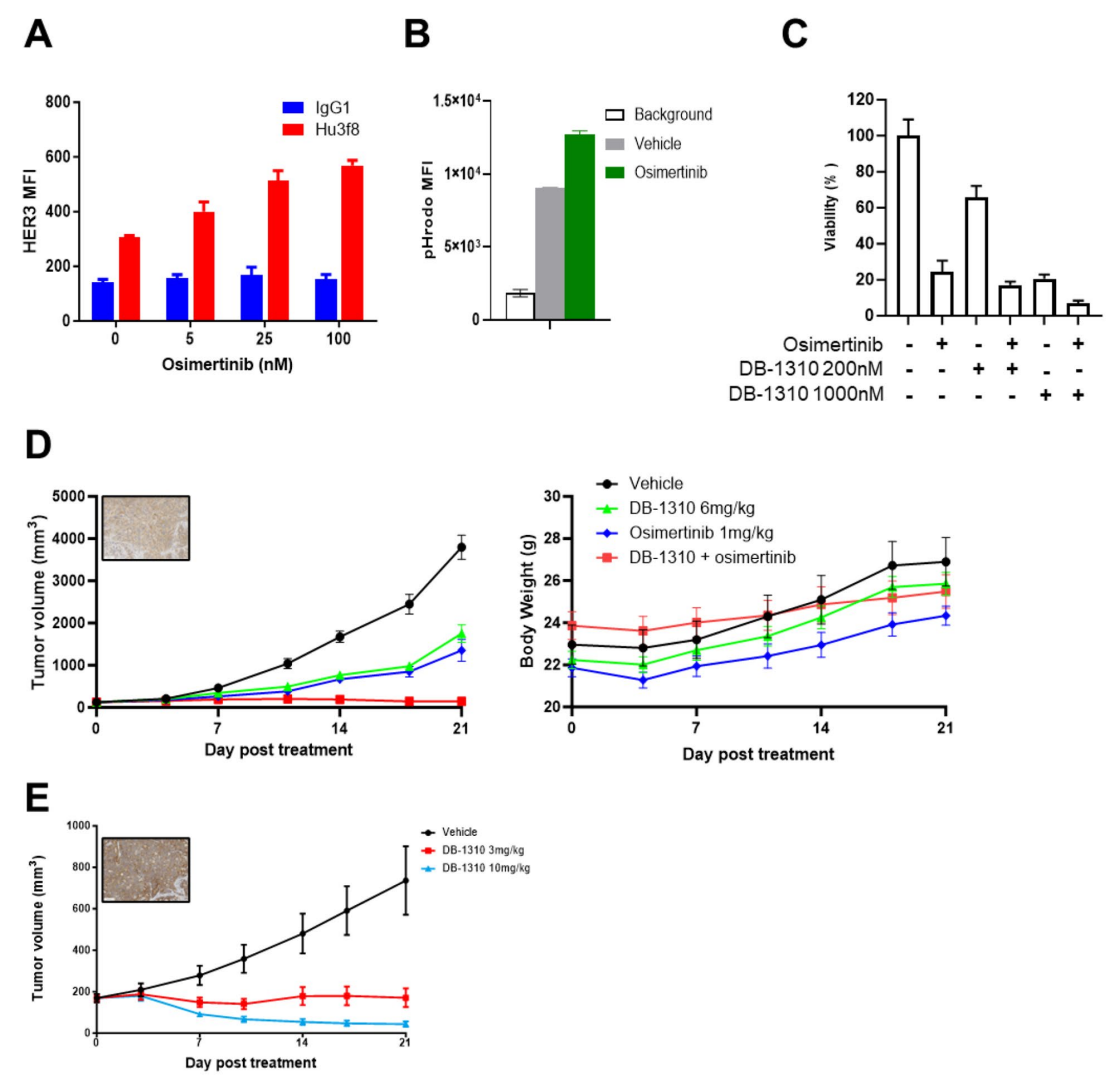
DB-1310 suppresses EGFRm NSCLC tumor growth. (**a**) Surface HER3 expression on NCI-H1975 NSCLC cells was measured by FACS after osimertinib stimulation for 24 h. (**b**) NCI-H1975 cells were treated with vehicle or 25 nM osimertinib for 24 h. HER3 internalization was measured by treatment with pHrodo-labeled DB-1310. (**c**) NCI-H1975 cells were treated with 25 nM osimertinib with or without DB-1310 at 200nM or 1000nM for 5 days. Cell viability was measured by a CTG assay. (**d**) NCI-H1975 tumor cells were inoculated into BALB/c nude mice, and the mice were treated with DB-1310 (i.v. one treatment) and/or osimertinib (p.o. daily). Tumor growth and body weight were measured. (**e**) An osimertinib resistant PDX model (LD1-0025-200717) was established with samples from osimertinib-refractory NSCLC patients, and the mice were treated with DB-1310.

HER3 expression is compensatory upregulated after EGFR signaling blockade [25, 26]. HER3-DXd suppresses the growth of EGFR-TKI-resistant NSCLC tumors via a mechanism independent of tyrosine kinase inhibition [12, 13, 25]. Thus, we tested whether DB-1310 can suppress the growth of EGFRm NSCLC tumors. NCI-H1975 cells are a human NSCLC cell line harboring the L858R/T790M EGFR mutation [27] and sensitive to osimertinib treatment. We tested proliferative inhibition of DB-1310 to NCI-H1975 both in vitro and in vivo. Cell surface HER3 expression and DB-1310 internalization were increased after osimertinib treatment (Fig. 5a and b). DB-1310 suppressed NCI-H1975 cell proliferation both in vivo and in vitro. The tumor suppression was further enhanced in the DB-1310 and osimertinib combinational treatment group, with greater maximal growth inhibition than in the single-treatment groups (Fig. 5c and d).

Acquired resistance to EGFR-TKIs therapy develops in NSCLC patients harboring EGFR mutations. The acquired EGFR C797S mutation was detected in osimertinib-refractory NSCLC patients and is considered the driver of EGFR-TKI resistance [28]. Therefore, we evaluated efficacy of DB-1310 in osimertinib resistant tumor model. DB-1310 suppressed tumor growth in a PDX model established with samples from osimertinib-resistant NSCLC patient harboring the EGFR Del19/T790M/C797S mutation (Fig. 5e). In summary, DB-1310 suppressed the growth of NSCLC tumors with and without osimertinib resistance. Combination therapy with DB-1310 and TKIs may further benefit NSCLC patients with EGFR mutations.

### Safety profile of DB-1310

In order to assess its safety profile for clinical, a 6-week repeated dose toxicity study of DB-1310 was performed in cynomolgus monkeys via intravenous administration once every three weeks for a total of three doses. All animals survived until scheduled necropsy, without significant abnormalities found upon clinical observation, body weight, food consumption, ophthalmological examination, and cardiovascular system. The highest non severely toxic dose (HNSTD) was considered to be 45 mg/kg, with lung and thymic toxicity noted as the main forms of toxicity observed (Supplementary Table 3). Those result from toxicologic findings suggest acceptable safety profile of DB-1310.

## Discussion

In the current study, we developed a HER3 ADC called DB-1310 using a novel antibody conjugated to our proprietary DNA topoisomerase I inhibitor payload. DB-1310 demonstrated antitumor activity in lung, breast, colorectal and prostate cancer models with better tumor suppression than HER3-DXd. This tumor inhibitory effect is mediated mainly through three mechanisms. The first is payload-mediated cytotoxicity activity. DB-1310 was efficiently up-taken by HER3 + tumor cells with lysosomal localization and payload release. It induced cell cycle blockage and cell growth inhibition in vitro. The increase of intratumor free payload concentration correlated with the induction of DNA damage marker following DB-1310 administration in xenograft model. Second, DB-1310 has a bystander antitumor effect, eliminating both target cells and surrounding tumor cells. Finally, DB-1310 exhibits ADCC activity. DB-1310 used a wild-type human IgG1 Fc region with high affinity for FcRs. Our results showed that DB-1310 elicits NK cell-mediated cytotoxicity, while HER3-DXd exhibits neither ADCC nor CDC activity [21]. This may contribute to DB-1310’s superior tumor inhibition in in vivo tumor models. In summary, we developed a novel HER3 ADC and demonstrated its antitumor activity in preclinical cancer models.

Anti-HER3 antibodies bind several epitopes to achieve tumor suppression through the inhibitory activity of the HER3 hetrodimerization or HER3-heregulin interaction [13, 29–31]. Patritumab induces HER3 internalization and also blocks ligand binding [13, 32]. 10D1F is another anti-HER3 antibody binding HER3 heterodimerization interface. It inhibits both ligand dependent and independent HER3 signaling activation with single-agent activity in tumor growth inhibition for both in vitro and in vivo. However, HER3 internalization was not observed after 10D1F binding [33]. DB-1310 binds HER3 via a new epitope, which is distinct from the epitope recognized by patritumab, inducing fast HER3 internalization. The binding with this novel epitope may facilitate fast up-taken of DB-1310 by HER3 + tumor cells with on-target delivery of the payload. The tumor suppression was not observed by Hu3f8 alone treatment. It’s uncertain whether DB-1310 also blocks heregulin-induced HER2 and HER3 dimerization and downstream signaling. We will identify the binding site of DB-1310 and clarify its impact in HER3 signaling as additional potential mechanism of antitumor activity in future studies.

DB-1310 was found to show synergistic effects on tumor inhibition with osimertinib in NSCLC model. It’s due to feedback on HER3 upregulation and internalization with blockade of EGFR signaling [25, 26]. The add-on of DB-1310 to the EGFR-TKI therapy for the treatment of EGFRm NSCLC patients may further enhance the efficacy. In addition, DB-1310 suppressed tumor growth in a NSCLC PDX model established with samples from osimertinib therapy-refractory patients. HER3 overexpression and payload-mediated tumor suppression is independent to resistance mechanism to EGFR TKIs [5, 11–13]. DB-1310 demonstrated tumor-suppressive activity against HER3-expressing tumors, indicating that it could be new therapeutic selection for the EGFR-TKI refractory NSCLC patient. We will explore the therapeutic efficacy of DB-1310 in NSCLC patients with EGFR active mutation in the future.

Dose limiting toxicity is a key challenge for ADC development. Even the mechanisms of ADC toxicity are not full clearly understood, the premature release of free payload in circulation and off-tumor uptake are considered as two major factors contribute to payload related toxicity [34]. The linker-payload platform used for DB-1310 development displayed improved ADC stability in plasma with less free payload release in circulation [19]. The homogenous conjugation and stable linker-payload technology would enhance the molecule stability of DB-1310 and reduce its premature payload release. The hematologic toxicity was the most common treatment-emergent adverse events in HER3-DXd clinical development [5, 10]. Decreased reticulocyte, lymphocyte and platelet counts were also observed in a preclinical nonhuman primate (NHP) toxicity study of HER3-DXd [21]. A decrease in the reticulocyte count was observed after treatment with DB-1310 at doses of 30 mg/kg or higher but was reversed after 4 weeks of drug discontinuation. This effect may be associated with the on-target–off-tumor activity of the HER3-targeting ADC, with DB-1310 showing comparatively less bone marrow toxicity. The inflammation in lung was one major adverse effects observed in the toxicity study of DB-1310. These typical payload-associated findings were generally minimal to moderate and showed reversibility after a 4-week recovery. The tissue cross reaction study also showed binding of DB-1310 to pneumocytes only in monkeys but not in humans (data not shown), indicating that humans may be more resistant to the pulmonary toxicity of DB-1310 than monkeys. On the basis of preclinical evaluation, the first in human open-label phase 1/2a clinical trials of DB-1310 are currently underway in advanced solid tumors (ClinicalTrials.gov identifier: NCT05785741). It aims to assess the safety, tolerability, pharmacokinetics, and preliminary antitumor activity of DB-1310 in subjects with advanced/unresectable, or metastatic NSCLC with or without EGFR active mutation, HER2 positive breast cancer, castration-resistant prostate cancer, head and neck squamous cell carcinoma. HER3 expression in tumor and other exploratory markers will be also explored in this study. We may further adjust these development directions per accumulating data and new treatment landscape changes.

## Conclusions

In summary, DB-1310 demonstrated potent antitumor activity through multiple mechanisms with a safety profile. It can also enhance the tumor growth inhibition of EGFR-TKi for NSCLC patients. Furthermore, the tumor suppressive activity of DB-1310 is independent to the EGFR mutation and sensitivity to EGFR-TKi. DB-1310 may be a promising treatment option for patients with HER3 + tumors with further assess of safety, tolerability, pharmacokinetics, and preliminary antitumor activity in phase 1/2a clinical trials.

### Electronic supplementary material

Supplementary material

## Data Availability

The data that support the findings of this study are available from the corresponding author upon reasonable request.

## Abbreviations

ADC: Antibody–drug conjugates
ADCC: Antibody-dependent cell-mediated cytotoxicity
AUC: Area under the curve
CDC: Complement-dependent cytotoxicity
CDX: Cell line-derived xenograft
CTG: CellTiter-Glo luminescent cell viability
DAR: Drug-antibody ratio
DS-8201: Trastuzumab deruxtecan
EC50: Half-maximal effective concentration
EGFRm: EGFR-mutant
EGFR-TKIs: EGFR tyrosine kinase inhibitors
HER3-DXd: Patritumab deruxtecan
HNSTD: Highest non severely toxic dose
Hu3f8: Humanized 3f8 antibody
mAb: Monoclonal antibody
MFI: Mean fluorescence intensity
NHP: Nonhuman primate
NSCLC: Non-small cell lung cancer
PDX: Patient-derived xenograft
PK/PD: Pharmacokinetic/ pharmacodynamic
P-H2AX: Phosphorylated Histone 2AX
SPR: Surface plasmon resonance
TGI: Tumor growth inhibition rate
