# Supplementary material for: DB-1310, an ADC comprised of a novel anti-HER3 antibody conjugated to a DNA topoisomerase I inhibitor, is highly effective for the treatment of HER3-positive solid tumors
Source: J Transl Med. 2024 Apr 17;22:362. doi: 10.1186/s12967-024-05133-7 (PMC11022355; doi:10.1186/s12967-024-05133-7)
Supplement: Supplementary file 1 — Supplementary material [file 12967_2024_5133_MOESM1_ESM.docx]

**ADDITIONAL FILES**

**Supplementary Figure 1.** DB-1310 has a homogenous conjugation. **a.** The sequence and resides position of the DB-1310 parental antibody**. b.** Schematic of the structure of DB-1310. **c.** HPLC analysis of DB-1310 with display of conjugated heavy and light chains.

**Supplementary Figure 2.** Suppression of tumor growth by P1021, DB-1310 or Hu3f8. Cancer cell lines were cultured with P1021, DB-1310 or Hu3f8 antibody for 6 days. Cell proliferation was measured by a CTG assay (a), and IC50 values were calculated using linear regression with GraphPad Prism (b).

**Supplementary Figure 3.** DB-1310 induces Histone 2AX phosphorylation. NCI-H441 tumor-bearing mice were treated with 1 mg/kg or 5 mg/kg DB-1310. Tumors were harvested at the indicated time points, and the level of phosphorylated Histone 2AX was measured by western blotting.

**Supplementary Table 1** Affinity of DB-1310 and Hu3f8 for human and cynomolgus/rhesus HER3, as measured by SPR.

**Supplementary Table 2** Affinity of DB-1310 and Hu3f8 to Fcγ receptors and C1q, as measured by SPR.

**Supplementary Table 3** Summary of critical findings in 6-week repeated dose toxicity study with Cynomolgus monkeys.


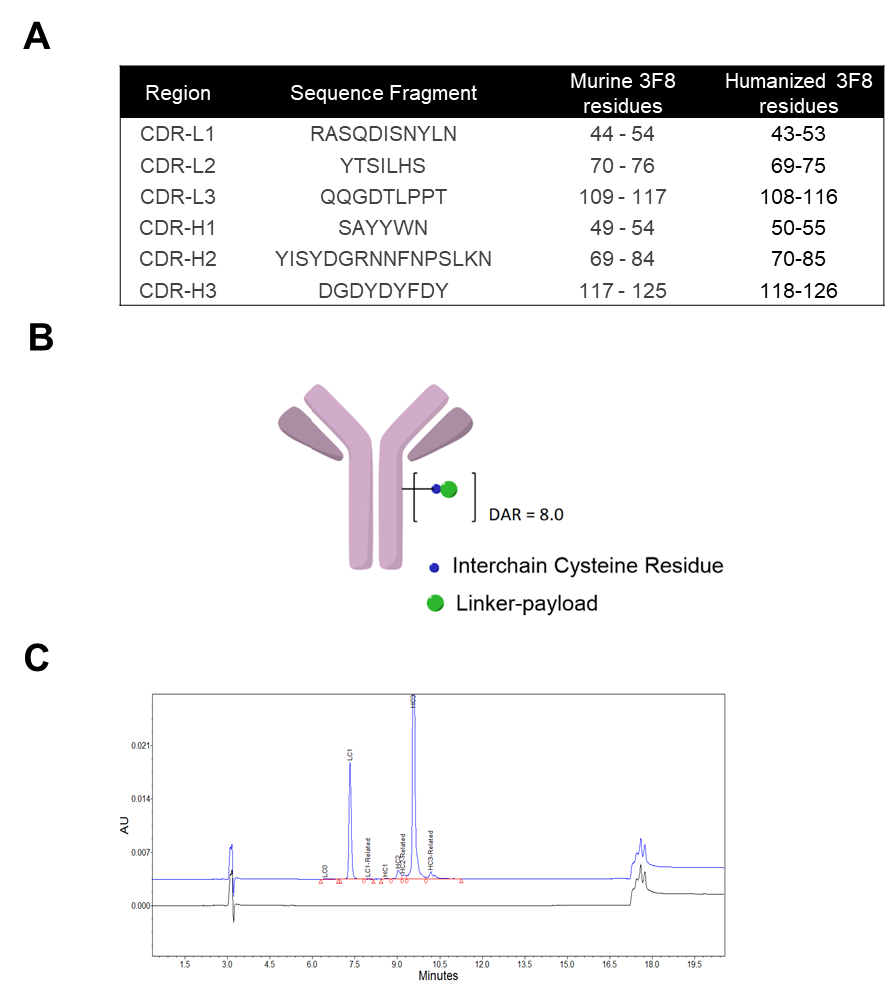


**Supplementary Figure 1**


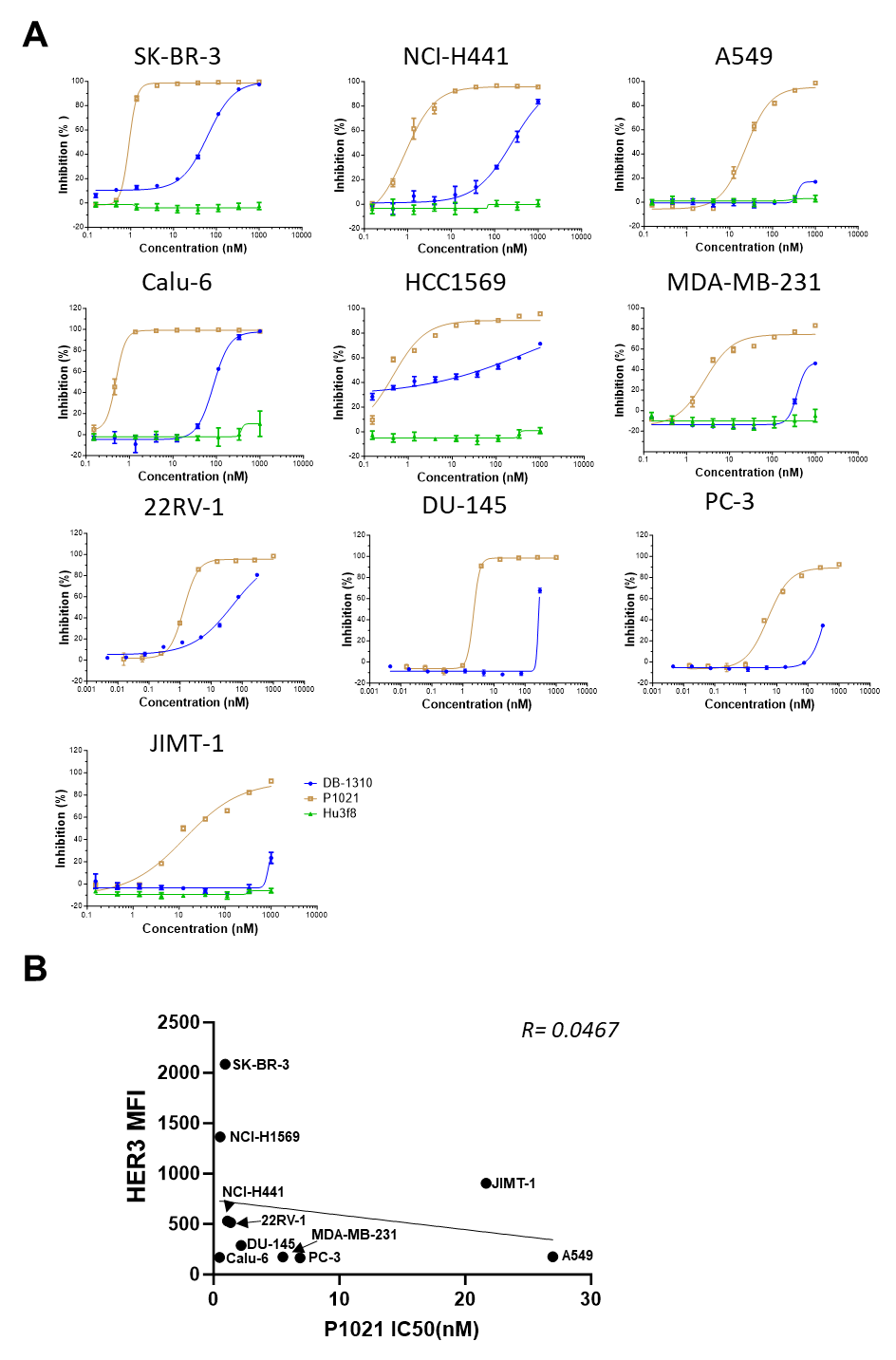


**Supplementary Figure 2**


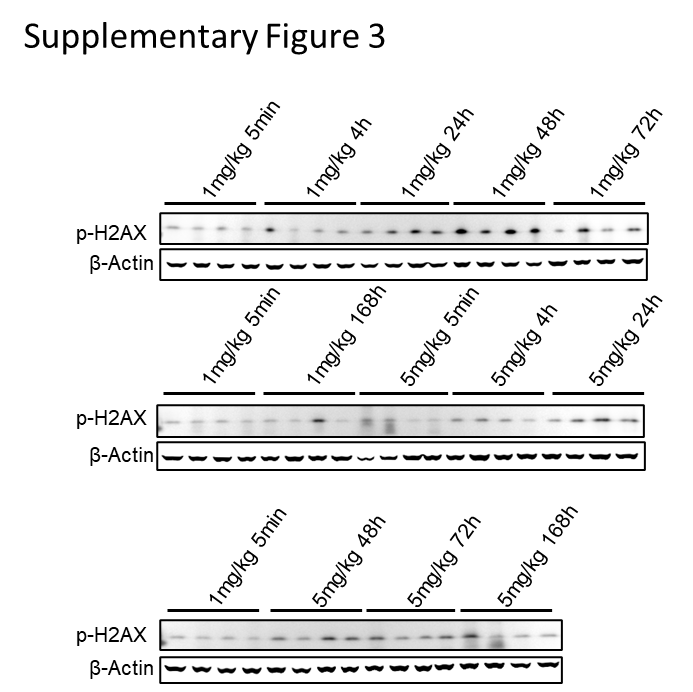


**Supplementary Figure 3**

**Supplementary Table 1** Affinity of DB-1310 and Hu3f8 for human and cynomolgus/rhesus HER3, as measured by SPR.

| Capture Ligand | Analyte | Ka | Kd | KD (M) |
| --- | --- | --- | --- | --- |
|  |  | 1/Ms | 1/s | M |
| Hu3f8 | Cynomolgus/Rhesus HER3 | 3.57 x 10^5^ | 3.01 x 10^-4^ | 8.43 x 10^-10^ |
| DB-1310 | Cynomolgus/Rhesus HER3 | 2.29 x 10^5^ | 2.91 x 10^-4^ | 1.27 x 10^-9^ |
| Hu3f8 | Human HER3 | 5.15 x 10^5^ | 3.09 x 10^-4^ | 6.00 x 10^-10^ |
| DB-1310 | Human HER3 | 5.21 x 10^5^ | 2.60 x 10^-4^ | 4.99 x 10^-10^ |

**Supplementary Table 2** Affinity of DB-1310 and Hu3f8 to Fcγ receptors and C1q, as measured by SPR.

| Capture Ligand | Analyte | Ka | Kd | KD (M) |
| --- | --- | --- | --- | --- |
|  |  | 1/Ms | 1/s | M |
| Human FcRn | Hu3f8 | N/A | N/A | 2.05 x 10^-7^ |
|  | DB-1310 | N/A | N/A | 1.95 x 10^-7^ |
| FcγRIIIA (F176) | Hu3f8 | 1.09 x 10^5^ | 8.16 x 10^-2^ | 7.50 x 10^-7^ |
|  | DB-1310 | 7.47 x 10^4^ | 9.52 x 10^-2^ | 1.27 x 10^-6^ |
| FcγRIIIA (V176) | Hu3f8 | 4.62 x 10^5^ | 2.62 x 10^-2^ | 5.68 x 10^-8^ |
|  | DB-1310 | 5.26 x 10^5^ | 4.10 x 10^-2^ | 7.78 x 10^-8^ |
| FcγRIIIB(NA1) | Hu3f8 | N/A | N/A | 1.73 x 10^-5^ |
|  | DB-1310 | N/A | N/A | 3.56 x 10^-5^ |
| FcγRIIIB(NA2) | Hu3f8 | N/A | N/A | 8.17 x 10^-6^ |
|  | DB-1310 | N/A | N/A | 2.52 x 10^-5^ |
| FcγRIIA(R167) | Hu3f8 | N/A | N/A | 9.13 x 10^-6^ |
|  | DB-1310 | N/A | N/A | 1.12 x 10^-5^ |
| FcγRIIA(H167) | Hu3f8 | N/A | N/A | 3.99 x 10^-6^ |
|  | DB-1310 | N/A | N/A | 6.23 x 10^-6^ |
| FcγRIIB | Hu3f8 | N/A | N/A | 3.01 x 10^-5^ |
|  | DB-1310 | N/A | N/A | 1.42 x 10^-5^ |
| FcγRI | Hu3f8 | 2.84 x 10^5^ | 1.70 x 10^-3^ | 5.99 x 10^-9^ |
|  | DB-1310 | 4.97 x 10^5^ | 1.61 x 10^-3^ | 3.24 x 10^-9^ |
| C1q | Hu3f8 | 2.08 x 10^7^ | 7.70 x 10^-3^ | 3.70 x 10^-10^ |
|  | DB-1310 | 3.19 x 10^7^ | 9.42 x 10^-3^ | 2.95 x 10^-10^ |

**Supplementary Table 3** Summary of critical findings in 6-week repeated dose toxicity study with Cynomolgus monkeys.

| Regimens | 0, 10, 30 and 45 mg/kg |
| --- | --- |
|  | Intravenous infusion, once every three weeks for 6 weeks (3 times in total) |
| No. of animals | All dose groups:  3/sex/group (main);  2/sex/group (recovery); |
| Hematology | ≥ 10 mg/kg: decreased WBC, neutrocytes and lymphocytes;  ≥ 30 mg/kg: decreased RET; |
| Target organs and tissues | ≥ 30 mg/kg: thymus;  45 mg/kg: lung |
| HNSTD | 45 mg/kg |

WBC, white blood cells; RET, reticulocyte; HNSTD, highest non severely toxic dose.
